# Hematopoietic Stem Cell Mobilization With Plerixafor Is Safe and Effective in Poorly Mobilizing Acute Myeloid Leukemia Patients

**DOI:** 10.1097/HS9.0000000000000176

**Published:** 2019-02-08

**Authors:** Evgenyi Shumilov, Urban Novak, Barbara Jeker, Behrouz Mansouri Taleghani, Ulrike Bacher, Thomas Pabst

**Affiliations:** 1 Department of Hematology and Medical Oncology, University Medicine Göttingen (UMG), Göttingen, Germany; 2 Department of Medical Oncology, Inselspital, University Hospital Bern, University of Bern, Bern, Switzerland; 3 Center for Hemato-Oncology, University Cancer Center Inselspital, Inselspital, University Hospital Bern, University of Bern, Bern, Switzerland; 4 Department of Hematology, University Hospital Bern, University of Bern, Bern, Switzerland; 5 Center of Laboratory Medicine (ZLM), Inselspital, University Hospital Bern, University of Bern, Bern, Switzerland.

Autologous stem cell transplantation (ASCT) can be applied to consolidate first remission (CR1) in favorable/intermediate-risk acute myeloid leukemia (AML) patients.^1–2^ However, up to 20% of AML patients in CR1 fail to mobilize a sufficient number of peripheral blood stem cells (PBSC).^3^ We evaluated safety and effectiveness of adding plerixafor to continued G-CSF stimulation in 5 AML patients in CR1, who had failed to mobilize at least 10 CD34^+^ cells/μL in the peripheral blood. The patients received a single dose of 24 mg plerixafor intravenously 4 hours before apheresis. Subsequent PBSC collection was successful in all patients enabling them to proceed to ASCT, and all autografts were molecularly minimal residual disease (MRD)-negative. These data suggest that plerixafor added to G-CSF stimulation is effective in AML patients with otherwise failing stem cell PBSC mobilization.

In AML patients in CR1, high CD34^+^ cell counts harvested in a single apheresis or high percentages of CD34^+^ cells in the autografts are associated with adverse outcome.^4^ We and others demonstrated that high numbers of peripheral circulating CD34^+^ cells at PBSC collection predicted higher relapse risk, whereas delayed hematologic recovery after ASCT was associated with better survival rates.^5–8^ Accordingly, a decreased mobilization potential after induction chemotherapy can indicate a favorable course in AML patients, in contrast to, for example, myeloma patients undergoing high-dose chemotherapy (HDCT)/ASCT.^5–7,9^

Mobilization failure in AML patients in CR1 is so far poorly studied, and subsequent alternative strategies are limited to bone marrow (BM) harvesting with all its inconveniences. Moreover, physicians are reluctant to use the rescue CXCR4 antagonist plerixafor in AML patients given the possible mobilization of residual leukemic stem cells and the possibility to harvest mobilized leukemic cells.^10^ However, this conclusion is not based on clinical data in this situation. Accordingly, we investigated in this study the safety and effectiveness of adding rescue plerixafor in AML patients, which otherwise would have failed stem cell mobilization.

We studied 5 patients with therapy-naïve de novo AML, who received 2 cycles of induction chemotherapy at the University Hospital of Bern. All patients had achieved CR after the first induction cycle and were planned for consolidation with HDCT/ASCT based on their genetic risk profiles (Table 1). The second induction cycle comprised cytarabine and daunorubicin in all patients, and when BM assessment on day 18 confirmed remission, G-CSF was started on the first day of neutrophils rising >0.5 G/L. Stem cell collection was planned on the first day of peripheral circulating CD34^+^ cells exceeding 20/μL. However, all 5 patients failed to achieve at least 10/μL despite continued G-CSF stimulation and were considered mobilization failure.

**Table 1:**
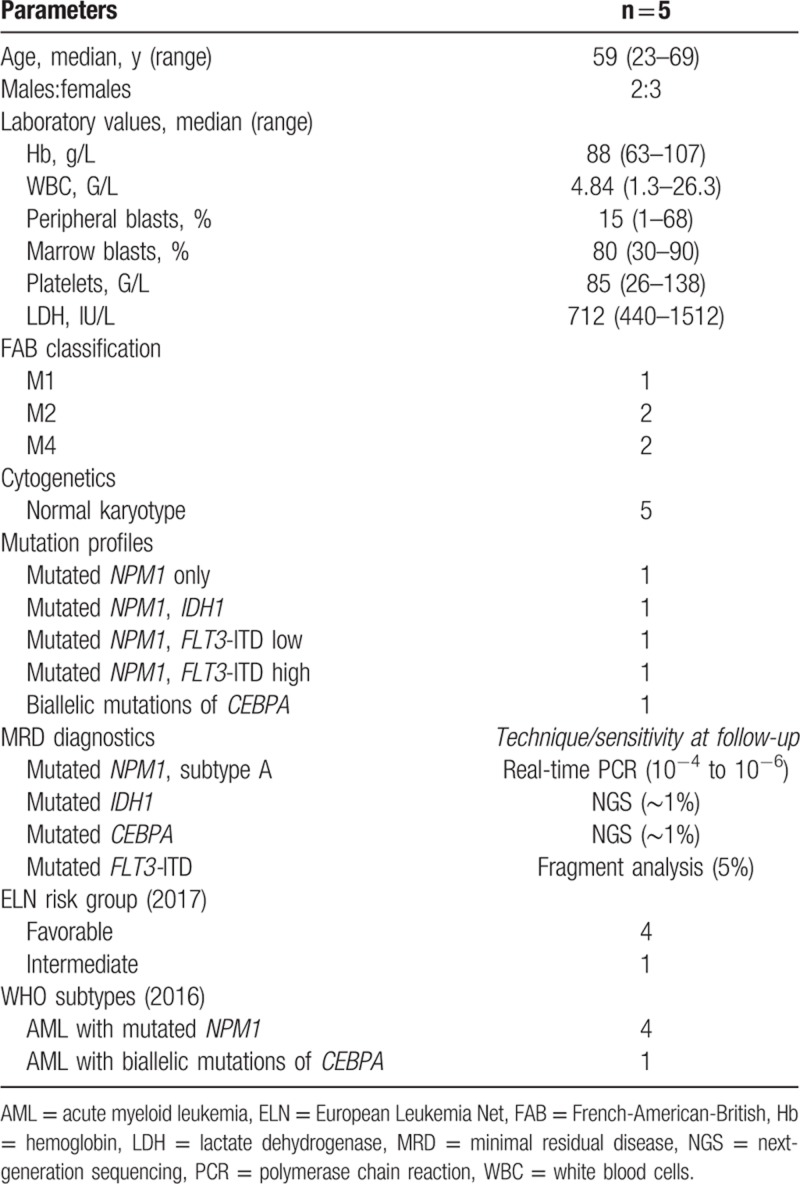
Clinical and Disease Characteristics at Diagnosis of 5 AML Patients With Imminent Mobilization Failure

According to European Leukemia Net criteria, 4 patients had favorable and 1 patient had intermediate risk.^11^ Four patients showed mutations in the *NPM1* gene, isolated in 1 case, or combined with an *FLT*-ITD in 2 patients (in the first one at a high allele ratio of 0.5; and in the second one at a lower ratio of 0.39), or *IDH1* (in 1 patient). The fifth patient had biallelic *CEBPA* mutations. All patients presented normal karyotypes. The patient with high *FLT3*-ITD allelic ratio and *NPM1* mutation underwent HDCT/ASCT due to primary biliary cirrhosis making this patient ineligible for allogeneic HSCT. MRD diagnostics were performed by real-time polymerase chain reaction (PCR) for *NPM1*, fragment analysis for *FLT3*, next-generation sequencing (NGS) for *IDH1*, and NGS and Sanger sequencing for *CEBPA*. Molecular MRD analyses indicated negativity both in the BM after second induction before SC collection and in the autografts.

The collection procedure in 3 patients was accomplished in a single day following plerixafor administration, whereas 2 patients needed 2 consecutive apheresis days with plerixafor given only at the first day. The median number of circulating peripheral CD34^+^ cells at the first day of PBSC collection was 3.8 cells/mL (range 1.6–6.0) before plerixafor, and it was 24.9 cells/mL 4 hours after plerixafor administration. The median harvest of collected CD34^+^ PBSC was 4.05 × 10^6^/kg (2.05–6.29), respectively (Table 2).

**Table 2:**
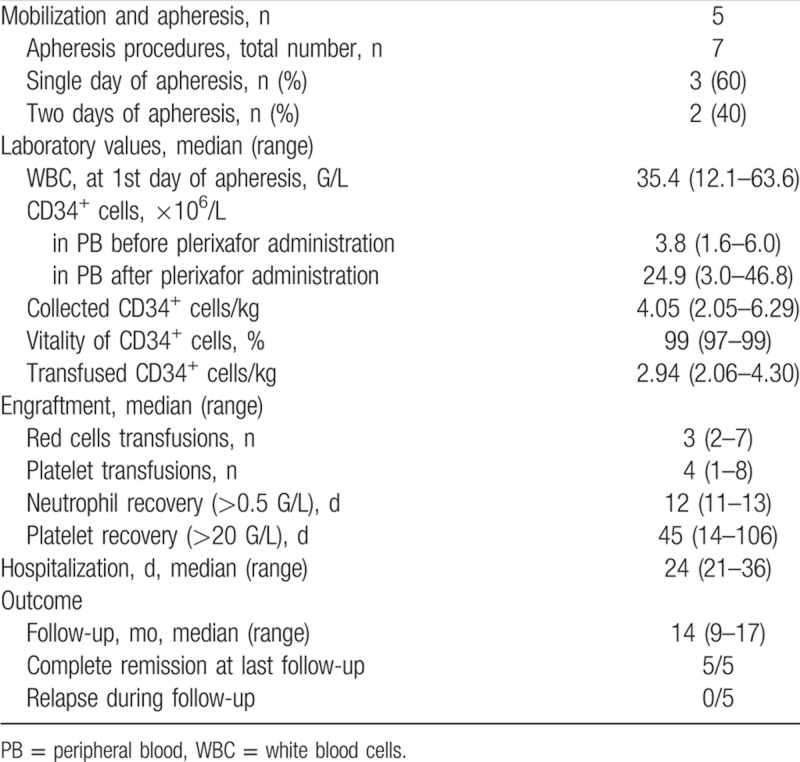
Mobilization of Peripheral CD34^+^ Cells and Stem Cell Collection in Acute Myeloid Leukemia Patients With Rescue Plerixafor Administration, Engraftment, Hospitalization, and Outcome

All patients undergoing HDCT before ASCT received full-dosed busulfan 4 mg/kg per day p.o. (days −6 to −3) and cyclophosphamide 60 mg/kg per day i.v. (days −2/−1), with PBSC reinfusion at day 0. A median of 2.94 × 10^6^/kg b.w. CD34^+^ PBSC was transfused (2.06–4.30 × 10^6^/kg). Patients received a median of 3 red blood cell transfusions and 4 platelet transfusions. Neutrophils recovered >0.5 G/L after a median of 12 days (11–13 days), and the median time until platelets increased >20 G/L was 45 days (14–106 days). All patients ultimately achieved complete hematologic recovery. The median hospitalization duration was 24 days (21–36 days). After a median follow-up of 14 months (9–17 months), all patients were alive in ongoing CR1.

Whereas available data suggest that ASCT with PBSC can be recommended to distinct subgroups of AML patients, there is little information on the mobilization failure rate and on rescue strategies for AML patients with a failed attempt of autologous PBSC collection.^12,13^ In AML patients, chemosensitivity of colony-forming units of granulocytes and monocytes derived from BM cells were described to correlate inversely with the peripheral maximum CD34^+^ cells peak during SC mobilization.^14^

Plerixafor, a small-molecule inhibitor of the CXCR4 chemokine receptor, has been approved in combination with G-CSF for PBSC mobilization for lymphoma and myeloma patients.^10^ However, plerixafor is so far not recommended for PBSC mobilization in AML patients due to the risk of mobilization of leukemic cells and potential autograft contamination. We report in this single-center study for the first time the safe and successful second-line mobilization of PBSC with plerixafor in AML patients who failed conventional mobilization with G-CSF after the second induction. Despite its limitations, our study suggests that the rescue administration of plerixafor induced significant additional mobilization of CD34^+^ cells from the BM to the peripheral blood, thereby allowing collecting sufficient CD34^+^ cells in all 5 patients. In fact, the median number of circulating peripheral CD34^+^ cells stimulated by G-CSF increased from 3.8 to 24.9 × 10^6^/L before and after plerixafor infusion. Consequently, plerixafor administration enabled these patients to proceed to subsequent consolidation with HDCT followed by ASCT.

Due to the potential of plerixafor for comobilization of leukemia stem cells,^8^ only MRD-negative patients combining different molecular techniques (Table 1) were selected for plerixafor application in this study, and MRD was excluded in the autografts. Acknowledging these strict conditions, all patients in this study maintained CR1 after a median follow-up of 14 months. Importantly, our data are limited to AML patients with MRD-negative CR1 in the BM and in the autografts as candidates for plerixafor administration after mobilization failure with G-CSF. Molecular MRD techniques should be comprehensive, including real-time PCR in the case of appropriate mutations, fragment analysis, and, increasingly, NGS.

Hematologic recovery after ASCT using plerixafor and G-CSF stimulation for collection of CD34^+^ PBSC is of obvious interest. Neutrophil recovery >0.5 G/L after ASCT occurred after a median of 12 days, and, thus, was identical as in a previous large study in AML patients receiving G-CSF only.^12^ Platelet recovery >20 G/L seemed prolonged in plerixafor mobilized AML patients, with a median of 45 days versus 16 days in G-CSF-only mobilized patients in our previous series.^12^ Possibly, this delayed platelet recovery in plerixafor mobilized AML patients reflects the background of primary mobilization failure and a poor stem cell pool in these particular patients.

In conclusion, rescue mobilization of PBSC with plerixafor was highly effective and safe in our small series of AML patients with primary mobilization failure. However, others have reported the development of secondary myelodysplastic syndromes or AML following rescue mobilization by plerixafor and subsequent HDCT/ASCT in 5 out of 43 patients with lymphomas or multiple myeloma after a median of 29 months after ASCT.^15^ Acknowledging the fact that these patients were heavily pretreated with 80% of them having received more than 2 prior chemotherapeutic regimens and with 20% having a history of previous radiotherapy, the question may arise whether plerixafor or rather the preceding intensive anticancer treatment truly contributed to the development of myeloid malignancies in these patients.^15^ Nevertheless, further studies should aim to better characterize the potential of plerixafor for reliable and safe PBSC mobilization combined with G-CSF in AML patients planned for ASCT.
